# Human Resource Practices for Corporate Social Responsibility: Evidence From Korean Firms

**DOI:** 10.3389/fpsyg.2022.893243

**Published:** 2022-04-07

**Authors:** Se-Rin Bang, Myeong-Cheol Choi, Ji-Young Ahn

**Affiliations:** ^1^School of Business, Ewha Womans University, Seoul, South Korea; ^2^College of Business, Gachon University, Seongnam, South Korea

**Keywords:** corporate social responsibility, external and internal CSR, person-organization fit, pay-for-performance, training and development, employee voice, labor productivity, employee retention

## Abstract

Human resource management (HRM) in managing environmental, social, governance (ESG), or corporate social responsibility (CSR) initiatives has been recently raised. Yet, little attention has been paid to integrating CSR and HRM. Our primary goal was to identify how and whether certain HR practices are critical for developing employee capability to operate in firms with active CSR initiatives. We first examine the impact of external CSR activities on firm-level work outcomes. Moreover, we attempt to identify a choice of particular HR practices that could be aligned with external CSR activities. We then empirically examine how each HR practice interacts with external CSR activities that influence employee retention and labor productivity. Using three longitudinal datasets conducted by the government-sponsored research institution over 154 publicly traded Korean firms for five waves of survey years, the results show that external CSR has a limited impact on employee retention and labor productivity. However, when external CSR activities are combined with a specific set of HR practices, including person-organization fit-based selection, performance-based pay, extensive investment of training and development, and employee suggestion program, the impact of external CSR on employee work outcomes is more substantial. The results indicate that external CSR and a particular set of HR practices as internal CSR can be complementary and generate a positive interaction on creating sustainable human capabilities.

## Introduction

There is a rapidly growing interest in how organizations benefit or harm social welfare. Corporate strategies or actions in this area are often referred to as environmental, social, governance (ESG), or corporate social responsibility (CSR). ESG represents how firms and investors integrate environmental, social, and governance concerns into their business models (Gillan et al., 2021). CSR has traditionally referred to corporate activities as more socially responsible and a better corporate citizen (Aguinis, 2011). One difference between the two terms is that ESG explicitly includes governance, and CSR indirectly includes governance issues related to environmental and social considerations. Therefore, ESG tends to be a broader term than CSR (Gillan et al., 2021). Vast literature attempts to find how CSR as corporate activity could impact a firm’s financial performance (Wang et al., 2016). However, prior research on CSR effects on firm performance has been characterized by conflicting or mixed findings at best (Margolis and Walsh, 2003; Ducassy, 2013; Wang et al., 2016). For instance, an empirical review of 127 studies by Margolis and Walsh (2003) suggested a positive relationship between CSR and firm performance through gaining market recognition. Similarly, Ducassy (2013) showed that 68% of the papers supported the positive link between CSR and financial performance, and 6% confirmed the negativity toward CSR and firm performance. Such mixed or conflicting empirical results may indicate that organizations differ in managing CSR strategies’ consequences.

Some studies attempt to clarify the relationship by emphasizing responses from specific stakeholders and CSR domains to solve such mixed results. For instance, studies have indicated the importance of employees’ responses from CSR activities because employees constitute important internal stakeholders who are central actors of CSR implementation, which can determine the effects of CSR on firm performance (Aguinis and Glavas, 2012; Farooq et al., 2017; Shen and Zhang, 2019). Moreover, emerging studies suggested employees may differentiate CSR initiatives based on various stakeholder groups instead of considering it as a unidimensional concept. Therefore, we divide CSR domains into external and internal CSR, followed by Farooq et al. (2017) and Deng et al. (2020). Specifically, external CSR is referred to as stewardship, including volunteerism and corporate philanthropy directed external stakeholders such as customers, business partners, and local communities. Internal CSR generally focuses on policies and practices of a firm that are related to the wellbeing of employees, their lives, and productivity (Farooq et al., 2017). It is predicted that external and internal CSR have a different impact on employees since the former has no direct gains on employees (Royle, 2005; Deng et al., 2020) while the latter is directed toward employees. Therefore, it is worth exploring how employees would respond to external CSR.

Following this research stream, this paper aims to investigate the impact of external CSR on work outcomes such as labor productivity and employee retention. Building on social identity theory, external CSR can promote positive employee attitudes and behaviors (Tyler and Blader, 2003; Valentine and Fleischman, 2008; Farooq et al., 2017; Deng et al., 2020). For example, studies have presented evidence supporting external CSR efforts improve perceived organizational prestige and higher self-esteem, positively related to employees’ job satisfaction, loyalty, and work engagement (Zhu et al., 2014; Deng et al., 2020). Therefore, it is expected that higher labor productivity could reflect all these positive attitudes and behaviors caused by external CSR activities.

Moreover, we argue that these external CSR and a specific set of HR practices as internal CSR can be complementary and positively affect sustainable human capabilities such as labor productivity and employee retention. HRM can be internal CSR itself that represents organizational actions to satisfy employees’ expectations, actively improve and fulfill organizational justice such as improving employee reward satisfaction, and ensuring work safety and the growth of employees (Greenwood, 2002; Crane et al., 2019). Building on the internal fit approach from strategic human resource management (SHRM) literature, we contend that employees respond differently to external CSR at different HR practices. Lastly, we attempt to identify the particular HRM practices to interact with external CSR within a firm and examine whether these practices can promote the effectiveness of external CSR initiatives.

This study contributes to the literature in two significant aspects: First, we extend our understanding of CSR-HRM link literature. Specifically, HRM has been treated separately from CSR as a cause or consequence of CSR (Aust et al., 2020). However, this study emphasizes the potential role of HR as internal CSR and the interaction between HR and external CSR. Building on the social exchange theory, employees are internal stakeholders of a firm that can be influenced by the actions such as HR practices. It is the norm of reciprocity between the employees and employer through the implementation of HR practices that focus on the wellbeing of employees (Low et al., 2017). Moreover, our study contributes sustainable HRM literature that still lacks much empirical attention to identifying the particular HRM practices (Macke and Genari, 2019; Aust et al., 2020). The study aims to empirically identify how and whether specific HR practices are critical for developing employee capability to operate in firms with active CSR initiatives.

## Theoretical Background and Hypotheses

### External CSR and Work Outcomes

As previous organizational psychology research suggested, we first revisit the relationship between external CSR and employee attitudes. Scholars have argued that external CSR can affect employees’ attitudes and behaviors by enhancing their organizational pride (Farooq et al., 2017; Deng et al., 2020). Based on social identity theory, it is expected that the employees tend to identify with an organization through comparison and association with other organizations to achieve self-worth improvement (Tyler and Blader, 2003). Specifically, employees can perceive more external CSR as a representation of the healthy image and reputation of the firm, which can be used to predict improvement of employees’ self-worth (Tyler and Blader, 2003; Farooq et al., 2017). Then, employees can earn a high level of organizational pride by comparing with external CSR of other firms (Valentine and Fleischman, 2008; Farooq et al., 2017). Such organizational pride can meet social identity needs, retaining employee engagement (Hogg and Turner, 1987). Empirical evidence suggests that external CSR can affect employees’ attitudes and behaviors through acquiring employees’ self-esteem promotion and organizational identification. For example, Jones and Kramar (2010) found that CSR can affect employees’ pride and identification with their organization, influencing organizational commitment and employee satisfaction. In addition, Rupp et al. (2018) found that work engagement is positively associated with CSR since employees are likely to be prouder of the firm. Studies have also presented that employees tend to increase a high level of self-worth evaluation and their attachment to the organization when a firm has a healthy reputation outside the organization (Peterson, 2004). These studies explain employee work engagement, enhancing positive attitudes and behaviors.

Furthermore, firms with high external CSR are expected to gain higher financial benefits. Deng et al. (2020) argue that external CSR initiatives are considered effective ways to manage relationships with other critical external stakeholders such as customers, government, and investors. Therefore, a firm’s investment in external CSR may reap the cost from receiving resources and support from the government, investors, and customers (Barnett and Salomon, 2006; Deng and Xu, 2017). Thus, employee work engagement outcomes, including employee retention and labor productivity, could be expected to present organizational pride, self-esteem promotion, and employee expectation from gaining financial benefits from external CSR.

To summarize, we extend prior research to revisit the relationship between external CSR and employee outcomes reflected by a reduced turnover and improved labor productivity to examine whether an organization can enjoy benefits from employees’ perceived external CSR. Put differently, the supposed positive association between external CSR and employee work outcomes, including labor productivity and employee retention, may indicate a form of strategic investment that positively affects firms’ economic outcomes and the creation of a “sustainable competitive advantage” (Su et al., 2016). This argument leads to the following hypotheses.

*H1a*: The external CSR activity is negatively related to employee turnover.

*H1b*: The external CSR activity is positively related to employee productivity.

### External CSR, HR Practices, and Work Outcomes

We further explore the possibility that the effect of external CSR on employee work outcomes may be contingent on HR practices as internal CSR in organizations. Emerging studies explore the possibility of interaction between external and internal CSR. For instance, Story and Castanheira (2019) showed that the interaction between internal CSR and external CSR increased employees’ organizational citizenship behavior. In addition, Deng et al. (2020) confirmed an S-shaped curve relationship between external CSR and labor productivity. Firms with higher internal CSR have a positive moderating effect in the S-shaped curve relationship. A study of the luxury business industry by Sipilä et al. (2021) found that external CSR activities negatively affect a company’s financial performance and customer loyalty. Internal CSR alleviated the negative relationship between external CSR and customer loyalty. Empirical studies in the hotel industry in Korea found that employee perception of internal CSR was more strongly related to pro-social behavior than external CSR perception. This positive relationship was more pronounced when external CSR perception was lower (Hur et al., 2020).

We argue that certain HR practices are highly related to internal CSR that can be better matched with external CSR activities, leading to higher employee work outcomes. Internal CSR and certain HR practices have in common that can influence the attitudes and behaviors of employees through the principle of reciprocity from social exchange theory (Luo and Zheng, 2013). The principle is defined as the human need and tendency to give something back when something is received. This need is more substantial when the gift is given without expecting a return (Slack et al., 2015). The reciprocity is particularly applicable to specific HR practices because they lead to some firms acting to support employee wellbeing and sustainability. Therefore, employees may feel obliged to pay these investments back by putting more effort or work engagement to reciprocate to firms (Slack et al., 2015; Aju and Beddewela, 2020). For instance, based on reciprocity, employees work harder or better to reward the organization by enhancing commitment and trust when employees receive fair wages (Luo and Zheng, 2013).

In this vein, certain HR practices as internal CSR can serve as a proxy for mutual support and trust, fulfilling employees’ work engagement (Tyler and Blader, 2003; Rupp, 2011; George et al., 2020). Thus, we contend that particular HR practices primarily take on ESG’s social aspect (S) and can be managed as internal CSR, creating positive synergies with external CSR. Part of the reason may be that mutual trust or reciprocity, combined with organization pride through external CSR, can create positive interaction on work engagement (Rupp, 2011; De Kock, 2021). Furthermore, anecdotal evidence suggested that when HRM and CSR managers agree on a mutual role in responsibility, the organization will act faster (Guerci and Pedrini, 2014). In addition, it seems probable that employees can perceive the firm’s effort to external CSR as mere construction of corporate images. This perception is highly likely when organizations lack internal CSR. Specifically, when employees feel that such internal CSR is insufficient, employees are likely to perceive external CSR activities negatively. It is consistent with recent studies arguing that firms exclusively focus on external CSR, and the outcome may be harmful due to employee cynicism (Low et al., 2017).

Furthermore, we argue that the internal fit perspective can explain the interaction between external CSR and HR practices. The perspective suggests that organizations need to seek efficient human resource practices, being dependent on the other strategic actions such as external CSR actions to obtain a “fit” or “internal consistency” between practices (Delery and Roumpi, 2017; Wright and Ulrich, 2017). The nature of the relationship that presents internal fit is complementary or synergistic between practices by mutually supplying each other’s lack that generates better performance than when each practice works in isolation (Wright and Ulrich, 2017). In addition, SHRM literature also views the particular “bundles” of HR practices that could make to organizational performance (Delery and Doty, 1996; Macky and Boxall, 2007). Specifically, these bundles consist of HR practices consistent with each other, such as selective recruiting, employee development, performance managing pay system, and employee involvement (Wright et al., 2003; Boxall and Macky, 2009; Wright and Ulrich, 2017). Following Wright et al. (2003), we divided multiple HR practices into four major functional areas in HRM: hiring, training, compensation, and employee participation. Then, we attempt to identify a particular HR practice from each functional HR area well aligned with CSR initiatives, aiming to create capabilities required for positive work outcomes.

#### Person-Organization Fit-Based Selection

The dominant approach in hiring employees in practice has been the person-job fit (P-J fit), referred to as selecting employees based on their knowledge, skills, abilities, and experience that a job is required (Kristof-Brown et al., 2005). However, recent studies have argued that the person-organization fit (P-O fit) approach has been gained much attention and has become an essential criterion for employee selection. P-O fit-based selection suggests that employees tend to be attracted and selected to an organization that shares similar values and goals as the organization (Coldwell et al., 2008). In addition, prior studies have confirmed that P-O fit-based selection positively impacts employee socialization, job attitude, employee behavior, and actual retention (Kristof, 1996). Later, several scholars, including Hoffman and Woehr (2006), suggested that the relative importance of these two types of fits depends on the context of the job task and the organizational purpose. We posit that P-O fit-based selection is better matched with external CSR activities, leading to higher employee work outcomes for the following reasons.

First, P-O fit-based selection in firms with a high-level external CSR is more likely to consider CSR values of individual employees during the selection process. Therefore, it may increase the chance of hiring employees who have a more favorable attitude toward CSR, promoting the effectiveness of the firm’s external CSR activities. For example, Hur and Kim (2017) suggested that the employees identify with a company that implements CSR initiatives, particularly when their values are aligned with the firm’s CSR initiatives. Second, selection based on P-O fit can improve communication and encourage helping behaviors by having similarities in values and attitudes (Kristof, 1996). According to the similarity-attraction theory, similar attributes between employees can create closer relationships (Moreland, 1985). Therefore, a higher level of similarity between organizational members based on P-O fit selection and attitude reduces role ambiguity and conflict levels, promoting cooperation and communication (Meglino et al., 1989). Such cooperation and lateral communication are mainly related to reciprocity and social exchange when organizations make more significant efforts when devising and implementing external CSR activities.

Similarly, Evans and Davis (2005) also revealed that selection based on P-O fit positively impacts group shared mental models. This approach can influence positive organizational support, consideration, and social responsibility among employees required in most CSR firms. Additionally, Kim et al. (2010) have suggested the importance of value-fit in CSR firms, implying that congruence between employee personality and characteristics of CSR activities may create the employee perception of organizations as a responsible organization perceived as organization pride.

To summarize, P-O fit-based selection combined with employee pride through external CSR efforts are consistent and signify employee work engagement. As proposed above, P-O fit selection can benefit from screening out potential employees who are less favorable or less matched toward the firm’s external CSR efforts, thereby increasing the success of external CSR initiatives. In addition, value-fit achieved by P-O fit selection facilitates communication and a strong sense of organizational support, reinforcing employee perception of organizational pride (Lee et al., 2012). Finally, P-O fit-based employee selection is more beneficial in firms with more external CSR initiatives, indicating positive employee work outcomes. Thus, we formulate the following hypotheses.

*H2a:* The negative relationship between external CSR activity and the turnover rate is stronger when the selection is based on person-organization fit.

*H2b*: The positive relationship between external CSR activity and productivity is stronger when the selection is based on person-organization fit.

#### Performance-Based Compensation

Pay-for-performance is the most direct and visible signal to employees about how firms satisfy employee expectations and increase employees’ perception of organizational justice (Rynes and Gerhart, 2000). We argue that performance-based pay and external CSR can be complementary, creating a positive synergy on employee performance. First, performance-based compensation as an incentive alignment mechanism ties employee compensation directly to the firm’s strategic actions, including external CSR. It can enhance employees’ external CSR cognition and motivate employees to take more external CSR-oriented activities. Anecdotal evidence suggests that employees’ actions toward external CSR activities such as *pro bono*, voluntary work, green behavior, donations to nonprofit organizations, and community involvement are more recognized during the performance evaluation and reward design (Gelade and Ivery, 2003; Orlitzky et al., 2006). In addition, pay-for-performance, particularly collaborative rewards based on collective performance such as profit-sharing, affects employees’ perception of mutual support and communication. It is because collaborative pay-for-performance can increase cooperation and coordination among members (Harrison et al., 2002), prevent social loafing (Pearsall et al., 2010), and share information among members (Chen and Kanfer, 2006). These employee attitudes and behaviors raised by collaborative pay-for-performance can create employees’ perception of CSR-favorable climate in an organization well matched with external CSR actions (Harrison et al., 2002).

Furthermore, recent studies argue that firms that need external CSR programs should promote short-term financial performance by implementing employee performance-improving systems. Therefore, the organizations taking external CSR initiatives are more likely to adopt performance-based pay because it motivates employees to work harder and better through monetary incentives to improve their short-term financial performance. For example, Jones and Kramar (2010) conducted a qualitative study for Australian companies. They found that the degree to which a firm is involved in CSR for external stakeholders was positively associated with adopting a pay-for-performance. In sum, performance-based pay combined with external CSR is complementary, creating positive interaction through increased employees’ awareness and motivation of external CSR activities.

Consequently, the alignment will create a CSR-favorable climate and improve the short-term financial performance. Thus, a performance-based pay system combined with CSR can promote employee work outcomes. It leads to the following hypotheses.

*H3a:* The negative relationship between external CSR activity and the turnover rate is stronger when firms use a pay-for-performance system.

*H3b:* The positive relationship between external CSR activity and the productivity rate is stronger when firms use a pay-for-performance system.

#### Extensive Investment in Training and Development

Employee training and development enhance employee skills and behaviors and the motivation to apply those skills and behaviors at work (Pfeffer and Veiga, 1999). Studies show that the advancement of employees’ skills and behavior is a critical part of internal CSR and employee training and development opportunity is a service provision by organizations (Low et al., 2017). A firm’s continued growth depends on meeting the needs of employees through employee development and establishing a positive social exchange relationship, thereby creating a perception of employee obligation (Ferreira and de Oliveira, 2014). Then, employee obligation can influence employees to benefit the firm through better quality of work or extra-role behaviors. In addition, this investment in employee training and development can also affect the employee perception of being valued or self-worth improvement (Kuvaas and Dysvik, 2009), thereby increasing organization pride. Thus, it is expected that extensive investment in employee training and development, an essential part of internal CSR, may enhance employee obligation, self-worth improvement, and commitment to organizations as responsible organizations. Furthermore, the above process may evoke more external CSR activities because a high level of obligation and mutual trust caused by a significant investment in employee development can increase employees’ tendencies to help others, including customers.

Moreover, recent studies suggest that employee training and external CSR are tightly coupled through CSR training by infusing the firm’s CSR values directly to employees (Ellis, 2009). For example, Ellis (2009) emphasized that CSR training can enhance employees’ awareness of CSR and improve their engagement in external CSR activities. Similarly, Obrad and Gherheș (2018) also classified environmental and social activities within organizations that present social responsibility toward the firm’s stakeholders. A majority of these activities were professional development workshops and training programs.

Overall, significant investment in employee training and development is expected to provide more potential benefits to organizations that pursue a more active external CSR activity. Compared to those who do not, firms with relatively significant investments in CSR activities tend to have higher mutual trust, commitment to organizations, willingness to help other external stakeholders, and CSR awareness. Employee training and development combined with external CSR activities can positively impact employee work outcomes. The argument leads to the following hypotheses:

*H4a:* The negative relationship between external CSR activity and employee turnover is stronger with the investment in employee training and development.

*H4b:* The positive relationship between external CSR activity and employee productivity is stronger with the investment in employee training and development.

#### Employee Voice

In practice, employee voice is represented by openness to considerations such as grievance procedures, suggestion systems, counseling services, employee management councils, survey feedback, non-management task forces, question and answer programs, and ombudsman services (Spencer, 1986). In addition, several studies have insisted that employees positively perceive employee voice because it sends the signal to employees that their inputs are valued and they are valued members of the firm (Morrison and Milliken, 2000; Milliken et al., 2015). In contrast, the lack of concern for employees’ ideas or suggestions could translate into an employee perception that the firm is not using procedural justice, thereby generating negative behavioral consequences such as frustration, stress, low self-control, self-efficacy, and quitting (Cohen-Charash and Spector, 2001).

We contend that employee voice influences firm’s external CSR activities and is influenced by external CSR. It appears that employee voice, being a direct feedback communication, may promote employees’ awareness of a firm’s efforts to invest in external CSR and influence how they view their firm’s CSR activities. For instance, Kirat (2015) found that a firm’s relationships with external stakeholders may depend on its efforts to communicate with employees. Additionally, Rupp et al. (2006) noted that employees are less likely to internalize a firm’s CSR into their daily operation fully when employees are less committed to developing and implementing CSR efforts through employee participation or voice. Similarly, Young and Thyil (2014) also show that firms displaying congruence between communication and CSR activities can encourage connectivity, allowing employee participation and engagement. In addition, a recent study maintains that employees as vulnerable stakeholders are less capable of exercising a direct influence on firms (Civera and Freeman, 2019). Therefore, the employee voice function may be a mechanism that can help confirm that the external CSR activities are aligned with the ethical or fair treatment of employees. This employee voice can be legitimately handled by the representative function such as employee councils or labor unions. Indeed, the promotion of open interaction between employees and their representatives and the participation of employee representatives in the firm’s decision-making process has been identified as pivotal parts of firms with active CSR efforts (Diaz-Carrion et al., 2019; Yu et al., 2021). Thus, the hypothesis is given as follows:

*H5a:* The negative relationship between external CSR activity and the turnover rate is stronger when firms use a suggestion system.

*H5b:* The positive relationship between external CSR activity and employee productivity is stronger firms use a suggestion system.

## Research Method

### Sample and Data

The study sample was constructed from three publicly available datasets in Korea. We used the five waves of CSR survey of 2010, 2012, 2014, 2016, and 2018, all of which were conducted by the Korea Economic Justice Institute (KEJI), and the HR data from the Human Capital Corporate Panel (HCCP), government-sponsored national employer survey in Korea. Financial data from the Korea Information Services (KIS) from 2010 to 2019 were also obtained. First, the KEJI index provides CSR scores for approximately 200 publicly traded firms in Korea. KEJI has published 200 CSR firms every 2 years and evaluated CSR scores in terms of seven components of CSR activities: environmental conservation community service, organizational integrity, justice, customer satisfaction, employee satisfaction, and economic development (Lee et al., 2017). Both quantitative and qualitative approach assesses each component. The quantitative methods use a wide range of archival sources, including firm annual reports; reports from the Fair Trade Commission in Korea, the newspaper article about illegal corporate activities; the Korea Employment Agency for the Disabled and the Korea Investors Service; and certifications from the Korean Agency for Technology and Standards and the Ministry of Environment (Lee et al., 2017). The qualitative method is conducted from a survey designed by professional researchers and representatives of civic groups.

Second, HCCP is employee-employer panel surveys to acquire HRM information, including the firm’s availability of the particular HR practices. The survey respondents were largely HRM and business strategy managers, each of whom responded to the items related to their specialization. Over 450 Korean publicly traded firms participated in the survey, and a panel investigation was conducted at biennial intervals. HCCP consists primarily of a corporate-wide survey (Enterprise Survey) and a survey of employees (Workers Survey), divided into a head office survey and a site survey. Since this study was analyzed at the firm level, we used data obtained from the corporate-wide survey. We used these five waves of panel dataset to acquire HR-related information but used all financial data from 2010 to 2018 from the KIS value. The five waves of survey years from the KEJI dataset included 180 firms. After excluding 22 companies with neither financial information nor HR variables, our usable sample was 154 firms that completed the survey.

### Measures and Analyses

#### Dependent Variable

We used two employee work outcome indicators as dependent variables. First, we measured employee productivity (or labor productivity) as the net sales per employee obtained from firms’ financial statements. We can collect firms’ financial reports and calculate their productivity using the KIS-value dataset. Through cost reduction and asset utilization, corporate financial performance improves profitability and asset return. The rate of return on invested capital is essential such as operating income, sales, and total profit. Although various financial indicators exist, sales and operating profit benefit from directly gauging the market’s reaction as a quantitative indicator of the profitability of businesses (Lee et al., 2017). We measure productivity as sales divided by the number of employees in the given year. Secondly, the firm-level turnover rate was measured by the voluntary turnover divided by the number of total employees in the year from the HCCP dataset.

#### External CSR

External CSR score is obtained from the KEJI index, published in 2010, 2012, 2014, 2016, and 2018. As noted above, total CSR activity was measured to elicit the seven items of the KEJI index listed below: organizational integrity, justice, community service, customer satisfaction, environmental conservation, employee satisfaction, and economic development. We exclude employee satisfaction from the CSR Index directed at internal company members. The combined values of the remaining items except the employee satisfaction are considered CSR for external stakeholders.

#### HR Practices

*P-O fit selection (POF) variable* was measured based on the choice of survey questions in HCCP. The question requires HR managers to choose from 14 responses on what they consider necessary in the recruitment process. We used the measurement tool implemented by Cable and Judge (1996). If the firm had a selection program that pursues person-organization fit, one and the other were coded 0. In the present study, the questionnaire presenting recruitment based on person-organization fit has been included in survey questions of HCCP since 2016, not from the beginning year of the sample. Therefore, the number of observations from the sample about recruitment in this study to test hypotheses 2a and 2b is smaller than other samples.

*The pay-for-performance* was measured by a pay-for-performance plan from the HCCP dataset used. If a firm had a pay-for-performance or merit-pay system, it was coded 1; otherwise 0. Moreover, we measured *the extent of investment in employee training and development* by the total money spent on training and development programs obtained from the firm’s financial report from the KIS dataset. Finally, a *suggestion system* in the firm captures the measure of employee voice. Dundon and Gollan (2007) examined the 18 case studies, explored the purpose and meanings of employee voice, and suggested two motives for establishing employee voice systems: to eliminate employee dissatisfaction; to capture suggestions to improve organizational performance. Therefore, the types of content voiced through the formal suggestion system ranged from dissatisfaction to suggestions for improvement and participation in decision making. Information on the *suggestion system* was obtained from the HCCP dataset. If a firm adopted the suggestion system, it was coded 1; otherwise 0.

#### Control Variables

Several variables are included to account for some variation in the dependent or independent variables. We included *firm size, firm age, industry fixed effect*, and *firm financial performance*. These variables are organizational characteristics that are closely related to or may influence a firm’s HR practices and CSR activities (Evans and Davis, 2005). Firm size was measured as the natural log of total employees. Firm age is the number of years from the founding year to the given period. Financial performance at the past year (t-1), such as ROA(t-1), is controlled due to its influence on the firm’s CSR strategies or HR policies (Lee et al., 2017).

In this study, we conducted panel data analyses. Panel data provide information on individual firm behavior across individual firms (*i*) and over time (*t*). The panel data we used are unbalanced since firms are not observed in all periods. Selecting the appropriate empirical model in panel data analysis is important to ensure the correct estimation. The STATA (18.0 version) was used in this study to validate assumptions based on the variables above. There are two models used to analyze panel data: fixed-effect model and random effect model. We used the Hausman test method to determine which model was more appropriate. As a result of the Hausman test, the fixed-effect model is more appropriate than the random effect model. Its value (prob>chi^2^ = 0.0002) is not within the significance level (1, 5%) and is rejected. Fixed effects regression was used to test all of our hypotheses. In addition, we conducted the Shapiro–Wilk normality test, indicating the normal distribution of data. The following is the empirical specification model used in this study.


$$\begin{array}{l} \ln({\mathrm{Productivity}}_{i,t\mathrm{+1}})=\beta_{1}\text{external CSRit}+\beta_{2}\mathrm{HR}{\mathrm{Practice}}_{it}+\\ \;\;\;\;\;\;\;\;\;\;\;\;\;\;\;\;\;\;\;\;\;\;\;\;\;\;\;\;\;\;\;\;\;\;\;\;\;\;\;\;\;\;\;\;\;\;\;\;\;\;\;\;\;\;\;\;\;\;\;\;\;{\beta_{3}\text{(external CSR}*\mathrm{HR practice)}}_{it}+\\ \;\;\;\;\;\;\;\;\;\;\;\;\;\;\;\;\;\;\;\;\;\;\;\;\;\;\;\;\;\;\;\;\;\;\;\;\;\;\;\;\;\;\;\;\;\;\;\;\;\;\;\;\;\;\;\;\;\;\;\;\;\beta_{4}({\mathrm{Controls)}}_{it}+\varepsilon_{it} \end{array}$$

## Results

Table 1 presents the descriptive statistics and correlations for variables used in the study.

**Table 1:** Descriptive statistics and correlations.

S. No.	Variables	Mean	SE	1	2	3	4	5	6	7	8	9
1.	External CSR	49.35	6.90									
2.	Employee productivity	815,426	1,183,162.	0.08^***^								
3.	Turnover rate	5.11	14.26	−0.002^**^	−0.081^***^							
4.	POF	0.36	0.48	0.1	−0.008	−0.081						
5.	Pay-for-performance	0.47	0.50	−0.050	0.098^***^	−0.044	0.233^**^					
6.	Expense of training and development *	506.44	1013.15	0.058	0.045^***^	−0.037	−0.049	0.201^***^				
7.	Suggestion system	0.82	0.38	−0.095	0.053^*^	−0.010^*^	0.241	0.082^*^	0.007			
8.	Firm size	1570.20	2986.13	0.056	0.091^***^	−0.102^***^	−0.014	0.032^**^	0.053^***^	0.034		
9.	Firm age	51.18	17.34	−0.080^*^	−0.027^**^	−0.024	−0.010	−0.073^**^	−0.130^***^	−0.021^***^	−0.099^***^	
10.	ROA	2.76	28.64	0.014	0.048^***^	−0.646^***^	−0.011	−0.044	0.037^†^	−0.012	0.046^***^	0.023

Performance-based pay (existence 1; nonexistence 0), suggestion system (existence 1; nonexistence 0), and ^*^unit: Thousand won.

† *p < 0.10*.

* *p* *< 0.05;*

** *p* *< 0.01;*

*** *p < 0.001*.

Tables 2, 3 present the results of the panel regression analyses. Table 2 shows the effect of the explanatory variables on the firm-level turnover rate. First, external CSR is negatively associated with the turnover rate (*β* = −1.95, *p* < 0.05) shown in column 2 of Table 2, but the negative relationship between external CSR and the firm-level turnover rate is not always statistically significant across different empirical models presented in Table 2. Specifically, the coefficients of external CSR are not statistically significant or marginally significant when the specific HR practices are added in the regression models shown in column 3, column 7, and column 9 of Table 2. It indicates a potential interactive relationship between external CSR and HR practices. Thus, we do not have much evidence that external CSR impacts the turnover rate.

**Table 2:** Results of regression analysis of the CSR, HR practices, and Turnover rate.

Variables	Dependent variable: firm-level turnover rate									
	1	2	3	4	5	6	7	8	9	10
Firm size (log)	−9.20***	−9.33***	−2.80**	−2.83**	−8.88***	−8.82***	−9.38***	−9.35***	−7.02***	−7.02***
Firm age (log)	−0.07	−0.13	−2.38*	−2.38*	−0.08	−0.08	−0.55	−0.57	−0.78	−0.77
ROA (log)	−2.31*	−2.29*	−1.34	−1.36	−2.29*	−2.28*	−2.27*	−2.24*	−2.51*	−2.51*
Industry dummy	Yes	Yes	Yes	Yes	Yes	Yes	Yes	Yes	Yes	Yes
External CSR		−1.95*	−0.5	−0.08	−1.71^†^	−1.15	−1.63	−1.12*	−1.78^†^	−0.97
POF			1.03	2.11*						
Individual performance-based pay					1.31	−0.36*				
The expense of training and development(log)							−1.76^†^	−1.13		
Suggestion system									−1.57	−0.77
External CSR* POF				−2.15*						
External CSR* Performance-based pay						−0.18*				
External CSR* Expense of T&D								−1.37^†^		
External CSR* suggestion system										−0.55*
*R* ^2^	0.22	0.23	0.17	0.17	0.22	0.22	0.23	0.24	0.21	0.21
Number of observation	372	372	119	119	365	365	356	356	296	296

*Coefficient is non-standardized *β*. All variables were changed to logged variables for reducing the degree of distortion*.

† *p* *< 0.10;*

* *p* *< 0.05;*

** *p* *< 0.01;*

*** *p < 0.001*.

**Table 3:** Results of regression analysis of the CSR, HR practices, and productivity.

Variables	Dependent variable: labor productivity									
	1	2	3	4	5	6	7	8	9	10
Firm size (log)	3.61***	3.73***	2.19*	2.23*	4.73***	4.76***	2.89**	2.49*	3.28**	3.54***
Firm age (log)	2.93**	3.25***	1.5	1.54	2.91**	2.93**	3.81***	3.85***	2.76**	2.54*
ROA (log)	3.43**	3.41**	0.08	0.11	2.71**	2.70**	3.32**	3.22**	2.84**	2.85**
Industry dummy	Yes	Yes	Yes	Yes	Yes	Yes	Yes	Yes	Yes	Yes
External CSR		4.39***	3.89***	3.85***	3.55***	2.28*	4.41***	5.17***	3.89***	4.17***
POF			0.3	−0.72						
Individual performance-based pay					2.89	0.9**				
The expense of training and development (log)							4.11***	5.63***		
Suggestion system									1.97*	3.55***
External CSR* POF				−0.08						
External CSR* performance-based pay						0.5				
External CSR* Expense of T&D								4.05***		
External CSR* suggestion system										3.30**
*R* ^2^	0.07	0.1	0.08	0.09	0.12	0.12	0.13	0.15	0.11	0.13
Number of observation	627	627	141	141	519	519	607	607	423	423

*Coefficient is nonstandardized *β*. All variables were changed to logged variables for reducing the degree of distortion*.

* *p < 0.05*,

** *p* *< 0.01;*

*** *p < 0.001*.

In contrast, the results find the strong positive effect of external CSR on firm-level labor productivity (*β* = 4.39, *p* < 0.001) shown in column 2 of Table 3. Moreover, this positive relationship remains robust across different empirical specifications in predicting labor productivity, even when included in individual HR practices. Therefore, external CSR does not directly impact the turnover rate, but it confirms the positive association between external CSR and labor productivity. The results indicate limited evidence supporting that external CSR is positively related to employee work outcomes.

Hypotheses 2, 3, 4, and 5 predict the interaction effects of external CSR and individual HR practice on employee work outcomes. First, we examined the interaction between P-O fit-based selection and external CSR. As shown in column 4 of Table 2, the coefficient of the cross-product interaction term is negative and statistically significant (*β* = −2.15, *p* < 0.05), indicating external CSR combined with P-O fit selection can decrease turnover rate, supporting Hypothesis 2a. However, it is worth noting that the effect of P-O fit selection on turnover is positive but combined with external CSR, the interaction term becomes negative, and the marginal effect of POF on turnover rate becomes negative (*β* = −2.15 + 2.11 = −0.04, *p* < 0.05). Additionally, external CSR itself does not significantly affect the firm-level turnover rate. Still, its impact is increased when accompanied by the firm’s utilization of P-O fit-based selection. Thus, the results support the interactive relationship between P-O fit-based selection and external CSR in decreasing turnover rate. In contrast, we do not find evidence supporting the interaction between external CSR and POF on productivity shown in column 4 of Table 3. Thus, limited evidence supports the potential alignment between P-O fit-based selection and external CSR.

Moreover, Hypothesis 3a and 3b posit the interactive effect of pay-for-performance and external CSR activities. As presented in column 6 of Table 2, the interaction term of external CSR and pay-for-performance on turnover rate was negative and statistically significant (*β* = −0.18, *p* < 0.05), indicating external CSR activities combined with pay-for-performance practices can decrease firm-level employee turnover, supporting Hypothesis 3a. However, the interaction term of external CSR and pay-for-performance on productivity is positive but statistically insignificant presented in column 6 of Table 3, suggesting no strong evidence that supports the complementary relationship between external CSR and pay-for-performance in influencing productivity.

The potential interactive relationship between external CSR and investment in training and development was supported as proposed by Hypothesis 4a and 4b, suggesting a good fit between investment in training and development and external CSR. The regression coefficients of the interaction term of external CSR and investment in training and development on turnover rate were marginally negative (*β* = −1.37, *p* < 0.10), and the interaction term of external CSR and investment in training and development on training and development productivity is significantly positive (*β* = 4.05, *p* < 0.001) as presented in column 8 of Table 2, 3. The results suggest that the higher investment in training and development, the more positive the external CSR will have on the employee outcomes. Finally, as with Hypotheses 4a and 4b, Hypothesis 5a and 5b were strongly supported. The regression coefficient of the interaction between external CSR and suggestion system on turnover rate was negative and significant (*β* = −0.55, *p* < 0.05). In addition, the interaction term of external CSR and employee voice has a positive impact on productivity. (*β* = 3.30, *p* < 0.001). It indicates that the complementary effect of employee voice and external CSR significantly impacts the turnover decrease and labor productivity.

## Discussion

Our first purpose in this study was to examine the impact of external CSR activities on firm-level work outcomes. Hypothesis 1a and 1b predicted the negative effect of external CSR on firm-level employee turnover and the positive effect on labor productivity. Based on the panel data of 154 publicly traded Korean firms obtained from the separate archival sources on CSR activities, HR practices, and financial data for five waves of survey years, we found limited evidence of the significant impact of external CSR on work outcomes. The results show that external CSR does not significantly impact the turnover rate when the specific HR practice is added. It seems probable that the HR effect on employee retention partially absorbed the CSR effect, and the interaction term of the two variables was positive and significant, suggesting the two complementarily interacted with each other. Still, it supports the positive relationship between external CSR and labor productivity, implying that firm can partially benefit from employees’ perceived external CSR. The result is consistent with recent findings by Deng et al. (2020), suggesting that building on social identity theory, external CSR had a positive economic gain through the increased labor productivity due to the enhancement of employees’ self-esteem from the firm’s external CSR activities. However, our findings indicate that the effect of external CSR on employee retention diminishes as we include some HR practices, implying the potential interaction between external CSR and HRM.

Our primary goal was to identify how and whether specific HR practices are critical for developing employee capability to operate in firms with active CSR initiatives. Following sustainable HRM literature, recent studies have attempted to identify socially responsible HR practices such as CSR training and reward based on employee volunteering (Shen and Benson, 2016; Clarke and Boersma, 2017). However, these practices are somewhat peripheral and do not represent the overall function of HR. In addition, it is challenging to generalize through empirical analysis because these are used only by a few organizations. Thus, we identified the particular HR practices *a priori* from four primary HR functions: P-O fit-based selection, performance-based compensation, extensive investment in training and development, and suggestion system. We also predict the synergistic effect between the particular HR practice and external CSR.

First, our findings indicate weak evidence supporting the potential interaction between P-O fit-based selection and external CSR predicted by Hypotheses 2a and 2b. It suggests that P-O fit-based selection is more beneficial in firms with high external CSR activities. The result is consistent with recent empirical evidence indicating that the perceived value-fit may explain why some applicants are attracted to organizations that engage in CSR (Shen and Benson, 2016; Shen and Zhang, 2019). However, limited evidence shows the potential fit between external CSR and pay-for-performance, supposed by Hypotheses 3a and 3b. The results imply that performance-based pay as a retention tactic can positively interact with external CSR by becoming aware of CSR activities and motivating CSR efforts through organization pride to retain employees. However, our results indicate that the interaction has no impact on labor productivity. It is partly because pay-for-performance in firms with external CSR efforts may not necessarily provide employees with incentives to work harder and better. The results are partially consistent with prior studies, indicating that the extent to which a firm is involved in CSR activities was significantly related to implementing a pay-for-performance system to retain employees (Jung and Kim, 2016).

Moreover, our results predicted by Hypotheses 4a and 4b indicate strong evidence of the positive synergistic effect of external CSR and the amount of money spent on employee training and development. In addition, the findings indicate that extensive investment in training and development in firms with a high level of external CSR may create a high level of mutual trust, commitment to organizations, and employees’ CSR awareness which can positively influence productivity and employee retention. Finally, the positive interaction between external CSR and suggestion system predicted by Hypotheses 5a and 5b was strongly supported. The results suggest that firms presenting consistency between open communication from voice mechanism and CSR activities can encourage connectivity and work engagement, consistent with Young and Thyil (2014).

Based on these results, this study makes the following contribution. First, the findings extend our understanding of sustainable HRM by identifying how and whether specific HR practices are critical for developing the employee capability required to operate in firms with active external CSR initiatives. Our view is consistent with prior studies positing that external CSR is viewed as an independent function in its own right (Shen and Benson, 2016). Our evidence suggests that specific HR practices take on the social (S) aspect of ESG through managing as one part of the internal CSR. Then, particular HR practices can emerge and facilitate the full exploitation of synergies between internal and external CSR efforts. Finally, our findings indicate that building on the social identity theory and social exchange, reciprocity combined with organization pride will positively influence employee retention and performance.

Moreover, this study highlights the impact of CSR activities on firm-level employee performance from a strategic perspective. Prior studies mainly focused on the impacts of overall CSR on employee attitudes and behaviors such as organizational commitment (Orlitzky and Swanson, 2008; Jones et al., 2017), organization identification, and extra-role helping behavior (Shen and Benson, 2016). Thus, our study attempts to link CSR activities and HRM into financial or bottom-line results by utilizing firm-level retention and labor productivity variables from the macro HR perspective.

Furthermore, our findings contribute to our understanding of managing CSR initiatives and human resources in practice. Our results empirically provide evidence that P-O fit-based selection, performance-based pay, extensive investment of training and development, and suggestion system align well with external CSR activities, promoting the effectiveness of CSR initiatives. Therefore, firms need to recognize the importance of managing HR practices internally to integrate them into CSR strategies. A set of mutually complementary HR practices can be used to promote the success of CSR activities in organizations.

## Conclusion

In conclusion, this research contributes essential insight to the CSR-HR literature from the SHRM perspective. Specifically, we recognized that the implementation of well-matched HR practices is consistent with core values embedded in the external CSR activities. In addition, employees’ perception of social identity and social exchange plays a critical role in affecting CSR-HR interaction. Therefore, the findings can help organizations make strategic HR management which can signify the CSR-HR interaction.

### Limitation and Future Research

This study is not without any limitation that suggests further study needs. First, this research used the dichotomous measure that indicated the existence of a formal HR practice. Therefore, it did not assess the level of usage of “pay for performance” practices and employee suggestion programs. Secondly, this study did not directly test the underlying process through which employees’ social identity and the perception of reciprocity can affect the interaction between external CSR and HR practices in promoting work outcomes. Some refinements can be made in future research by utilizing case studies and employee perception surveys to illustrate a more understanding of the relationship. For example, a multi-level approach may help relate CSR initiatives and HRM to employee responses.

Second, it is also worth noting whether our focus on Korean firms can limit the generalizability of the findings. CSR activities have become a common practice and are viewed as an essential device where management directs firms through changing environments worldwide (Crane et al., 2019). However, geographical context may matter as there is a great deal of variation in the regulatory environment regarding CSR. Thus, further research is necessary to verify that our findings are generalizable to other countries. Moreover, our empirical evidence does not indicate a reverse-causal relationship that posits more successful firms are more likely to use CSR activities and developed HR practices. However, our study calls for a further study investigating the long-term effect of CSR and its interaction with HR practices on organizational performance.

Finally, this study suggested that the HR practices such as selection considering person-organization fit, performance-based pay, extensive investment for training and development, and suggestion system must be aligned to external CSR. However, with the excepted practices mentioned above, future avenues of research may examine the effect of other HR practices, such as job design, employee socialization, performance evaluation, and working environment that can consider the full range of HR practices. However, this study is limited by using four practices due to data availability. Such limitation may leave room for future research that will explore a configuration of HR bundle or HR system that promotes the success of CSR initiatives.

## Data Availability Statement

The raw data supporting the conclusions of this article will be made available by the authors, without undue reservation.

## Author Contributions

J-YA and S-RB contributed to the conceptualization, methodology, investigation, and writing—original draft. M-CC and J-YA participated in the manuscript revision, review, editing, and validation. All authors performed the data collection, data curation, and formal analysis and have read and approved the final manuscript.

## Funding

This work was supported by the Ministry of Education of the Republic of Korea and the National Research Foundation of Korea (NRF-2019S1A5A2A01050740).

## Conflict of Interest

The authors declare that the research was conducted in the absence of any commercial or financial relationships that could be construed as a potential conflict of interest.

## Publisher’s Note

All claims expressed in this article are solely those of the authors and do not necessarily represent those of their affiliated organizations, or those of the publisher, the editors and the reviewers. Any product that may be evaluated in this article, or claim that may be made by its manufacturer, is not guaranteed or endorsed by the publisher.
